# The Age of Artificial Intelligence in Neurologic Education: Where Are We Going With Educational Technology Today?

**DOI:** 10.1212/NE9.0000000000200093

**Published:** 2023-09-25

**Authors:** Roy E. Strowd

**Affiliations:** From the Department of Neurology, Wake Forest University School of Medicine, Winston Salem, NC.

Technology has influenced teaching and learning for centuries. Historically, educational technology was viewed as a tool, device, or platform used to deliver information.^1^ Physical slide projectors evolved into PowerPoint and software that rapidly present visually appealing data. In recent decades, e-learning and mobile-friendly teaching have expanded education globally and allowed educators to deliver instruction just in time for practice. Simulation technology has been incorporated as a tool to put learners in the driver seat, simulate patient safety events, evaluate effectiveness of educational interventions, and engage students in active learning. In the midst of these trends, the coronavirus disease 2019 pandemic catalyzed even wider-spread adoption of asynchronous learning and online communities of practice.^2^ Technology has facilitated an information age with rapid delivery and accumulation of data perpetuating memorization-based approaches to education.

Increasingly, medicine is moving away from the information age to an era of artificial intelligence (AI). Available medical knowledge exceeds learners' capacity to acquire and maintain it. Unfortunately, medical training continues to be marked by knowledge-based examinations, US Medical Licensing Examination steps, and board passing rates. The skill set needed for physicians in the future will be to manipulate new technology, collaborate with AI, and deliver patient-centered care using data monitoring systems in real time. Students need competence in using technology to sift through large amounts of data from varying sources, to critically appraise the veracity of information, to integrate this into their lifelong learning, and to share with a community of practice. Steve Wartman and others have called for learners to understand the 4 V's of big data instruction: volume (the vast amount of data), variety (data from different sources of varying validity), velocity (data generated rapidly), and veracity (the quality of data requiring differentiating information from misinformation).^3^

In this issue of *Neurology*® *Education*, a theme of technology-enhanced instruction emerges. Educators from across the training spectrum describe new approaches to using technology to enhance teaching and learning. There is much to be gained from these articles and yet still so much more for clinical neuroscience educators to tackle. In this editorial, I summarize key highlights, current gaps, and author opportunities for educators interested in pushing the boundaries for how educational technology can advance time-honored team traditions in the neurologic education fields.

## Overcoming Educational Gaps by Teaching Through Social Media

Social media can engage trainees in free, global, readily accessible, mobile-friendly learning. However, it is a tool with teaching challenges. The first 2 articles address several of these factors. In their study, Zelikovich et al.^4^ describe the results of implementing a virtual program for onboarding new neurologists to Twitter. The authors observed that asynchronous delivery of Twitter onboarding was feasible, but long-term behavior change was not. Even in these participants who expressed pressure to not miss out on important professional development, few engaged actively in social media in the long term. The second article by Alvarado-Dyer et al.^5^ describes a neurocritical care teaching curriculum delivered entirely through Twitter. Again, the authors demonstrate that knowledge can be disseminated, but long-term consistent behavior changes were “uncommon” and engagement varied.

What are educators to take away from these articles? Technology is a tool that when used to deliver information can break down geographic barriers, increase access, and overcome disparities, but may not cultivate curiosity without an intentional effort to do so. A growing body of research suggests that social cues and popularity play an important role in curiosity-driven learning.^6^ This is not inherent in social media, but can be harnessed when technology is used in the right way.

## Using Virtual Instruction to Standardize, Individualize, and Engage Learners

The next set of articles explore different approaches to using technology to teach the neurologic examination and emergencies. Several important themes on how to use technology in learning can be identified:

1. Standardization and Individualization: Reiter-Campeau et al.^7^ explore resident and faculty perspectives on teaching the neurologic examination virtually. They describe how virtual instruction can be used to standardize what is taught and individualize instruction for the learner. Virtual approaches can be an important tool for standardization and individualization.
2. Active Engagement: Ratliff et al.^8^ use podcasts before learning to incorporate experiential learning in resident conferences. Han et al.^9^ similarly show that residents overwhelmingly preferred active rather than passive instruction in the teleneurology examinations. Podcasts, simulations, and objective structured clinical examinations did not make learning active. Intentional use of technology is what activated learner engagement.
3. Learning to Learn: Both articles on teaching the neurologic examination highlight the challenges with virtual instruction and the value of using virtual approaches to teach the education process. Learning how to learn the neurologic examination proved as valuable as learning the techniques themselves.

## Where Are We Headed With AI in Neurologic Education?

Despite multiple articles on new technology, few high-quality studies have been received by *Neurology: Education* that address key research questions for AI. The journal is interested in articles on

1. AI in Education Research: How can AI be incorporated into teaching to improve learner outcomes? What outcomes are improved? How are they influenced by AI? What can we understand about theories of learning from implementing AI into instruction?
2. History of Neurology and Technology: What can we learn about implementing AI in education today by understanding how other technologies have been implemented and influenced instruction in the past? What themes can be drawn, and how can this be used by educators and scholars now?
3. Review of AI in Medical Education: How do educators and learners interact with AI in teaching, learning, and assessment? What does the data say about each of these areas of evidence-based education? How will ethics, privacy, transferability, and trustworthiness be navigated?

## A Turn Toward Applications, Admissions, and Affirmative Action

The last few articles in this issue focus on the fellowship application and interview process. Standardizing the application processes will foster greater equity by promoting transparency and access for all applicants. In this issue, the Viewpoint article by Geraghty^10^ calls for standardizing interview offer dates—the date that offers are released. The History of Medical and Neurologic Education reviews how standardizing the neuromuscular medicine fellowship time line increased transparency and fairness.^11^

These articles join the national debate on ways to promote equitable access to and greater diversity in educational institutions that has become even more relevant given this year's United States Supreme Court ruling on affirmative action. This ruling has had a substantial effect on undergraduate and professional school admissions and will likely influence application approaches to health care fields broadly. Clinical neurosciences face substantial barriers to recruiting qualified, diverse practitioners into the field. The supreme court decision creates new challenges to ensuring a diverse workforce at a time when studies clearly show that provider diversity improves patient care quality and outcomes.^12^

## AAN Virtual Recruitment Workgroup Consensus Statement for Residency Applicants

What about residency applications in neurology this year? The American Academy of Neurology has released a statement to guide applicants and programs. The AAN Virtual Recruitment Workgroup has released a consensus statement on the 2023–2024 application cycle. Guidelines for applying to neurology residency are in alignment with AAMC recommendations. See the accompanying *Neurology: Education* blog for links and more information.

Good luck to all learners and educators navigating the technology of the 2023–24 application season, and happy reading.
